# Applications using high-*T*_c_ superconducting terahertz emitters

**DOI:** 10.1038/srep23178

**Published:** 2016-03-17

**Authors:** Kurama Nakade, Takanari Kashiwagi, Yoshihiko Saiwai, Hidetoshi Minami, Takashi Yamamoto, Richard A. Klemm, Kazuo Kadowaki

**Affiliations:** 1 Graduate School of Pure & Applied Sciences, University of Tsukuba, 1-1-1, Tennodai, Tsukuba, Ibaraki 305-8571, Japan; 2 Division of Material Science, Faculty of Pure & Applied Sciences, University of Tsukuba, 1-1-1, Tennodai, Tsukuba, Ibaraki 305-8573, Japan; 3 Institute für Quantenoptik Universität Ulm, Albert-Einstein-Allee 11, D-89081 Ulm, Germany; 4 Department of Physics, University of Central Florida, 4000 Central Florida Blvd., Orlando, FL 32816-2385, USA

## Abstract

Using recently-developed THz emitters constructed from single crystals of the high-*T*_c_ superconductor Bi_2_Sr_2_CaCu_2_O_8+*δ*_, we performed three prototype tests of the devices to demonstrate their unique characteristic properties for various practical applications. The first is a compact and simple transmission type of THz imaging system using a Stirling cryocooler. The second is a high-resolution Michelson interferometer used as a phase-sensitive reflection-type imaging system. The third is a system with precise temperature control to measure the liquid absorption coefficient. The detailed characteristics of these systems are discussed.

Terahertz (THz = 10^12^ c/s) electromagnetic waves (EMWs) have various unique features that open new possibilities in the research areas of science and technology that enable various important applications, such as non-destructive evaluations, various imaging techniques, high-speed communications, security, medical diagnoses, biosciences, and biotechnologies, *etc*.^1^,^2^. From both technical and practical points of view, THz imaging may be the most widely demanded application, since THz EMWs can penetrate through many dry and nonmetallic materials as microwaves do, but their spatial resolution is better than microwaves due to their shorter wavelengths^3^,^4^,^5^,^6^. Another important potential field of applications may be the spectroscopic investigation and identification of particular molecules or polymers, which have definite fingerprint spectra due to molecular vibrations or rotations^7^,^8^,^9^.

Among the many imaging and sensing systems using THz EMWs that have been reported to date are a three-dimensional computed tomography (3D CT) imaging system^10^, a time-of-flight imaging system that can obtain depth information^11^, an imaging system below the diffraction limit using near-field lighting^12^, a THz chemical microscope^13^, and a high-resolution spectrometer using a THz-comb^14^. Systems based on THz time domain spectroscopy (THz-TDS) using pulsed-wave sources have made especially significant progress, and some systems have already been commercialized by Advanced Photonix Inc, Tera View, ADVANTEST Corp., *etc*. On the other hand, applications using continuous and monochromatic THz waves can be used for simpler and higher-speed imaging, imaging at a stand-off distance, higher-resolution spectroscopy, and coded information technology, *etc.* In order to put these applications to use, development of a compact, convenient, tunable, and powerful device is urgently needed.

In present THz imaging, backward-wave oscillators (BWOs) have been useful and very convenient THz sources with moderate power (mW) and frequencies ranging from a few 100 GHz to 1.5 THz^4^. Because BWOs employ vacuum electron tubes operating in a moderate magnetic field at room temperature, they have the advantage of emissions that are tunable by about ~100 GHz about their central frequency. On the other hand, BWOs have the disadvantages of their large masses (~50 kg), short operating lives (several hundreds of hours), power drifts over time, and rather poor reproducibility (≃1%), *etc*.^15^.

The unique (sub-) THz source based on a single-crystalline mesa structure of the high transition-temperature *T*_c_ superconductor Bi_2_Sr_2_CaCu_2_O_8+*δ*_ (Bi2212) was first reported in 2007^16^, and its subsequent development has proceeded rapidly^17^,^18^,^19^,^20^,^21^,^22^,^23^,^24^,^25^,^26^,^27^,^28^,^29^,^30^,^31^,^32^,^33^,^34^,^35^,^36^,^37^,^38^,^39^,^40^,^41^,^42^,^43^,^44^,^45^,^46^. Output radiation powers of a few tens of *μ*W have been consistently achieved from single conventional mesas^37^,^4^3,^44^, and of about 600 *μ*W was reported from a synchronized conventional three-mesa array^45^, and the radiation frequency could be varied almost continuously between 0.3 and 2.4 THz from a stand-alone mesa sandwich device^46^. In many experiments, the radiation frequency was measured using interferometer-based spectrometers^18^,^2^3–2^5^. Since optical path differences in these interferometers determine the spectral resolution of the spectrometers, it is commonly than 7.5 GHz, which is much larger than the spectral linewidth of the THz radiation from IJJ-THz emitters^35^.

The THz radiation from Bi2212 mesa structures discussed here is completely different from that of conventional semiconducting or optical devices in principle, and it has the characteristic features of continuous, stable, and monochromatic EMWs. In Bi2212 with the high superconducting *T*_c_ ~ 90 K, a periodic array of alternating superconducting CuO_2_ and insulating Bi_2_O_2_ double layers is stacked along the *c*-axis (perpendicular to the CuO_2_ plane) on an atomic scale. Each insulating layer behaves as an intrinsic Josephson junction (IJJ), which weakly couples the adjacent superconducting layers. Therefore, the mesa structure of Bi2212 consists of IJJs regularly stack of 653 junctions per 1 *μ*m stacked along the *c*-axis direction^47^. The fundamental mechanism of the THz EMW emission from the IJJ emitters was previously revealed to be the ac-Josephson effect^48^, with emission frequencies *f* satisfying *f* = *f*_J_ = (2*e*/*h*) (*V*/*N*), where *e* and *h* are the electronic charge and Planck’s constant, respectively, *V* is the applied dc voltage, and *N* in the number of active IJJs in the mesa. According to this relation, a dc voltage of 1 mV applied to each IJJ gives rise to an ac current with *f*_J_ satisfying the 2010 CODATA recommended value of 483.597870(11) GHz, Since *V* can be varied continuously in some ranges of the dc current-voltage (*I*-*V*) characteristic curve, the *f* can also be varied continuously. Therefore, tunable THz EMWs in principle can be generated by adjusting *V*.

However, the insulating layers in the mesa cause it to behave as an electromagnetic cavity, which allows for internal standing THz EMWs. When *f*_J_ matches a cavity resonance frequency *f*_cav_ of the particular mesa shape, the output power is significantly enhanced. For a rectangular mesa of width *w*, the observed *f*_cav_ = *c*_0_/(2*nw*), where *c*_0_ is the light velocity in vacuum, and *n* ~ 4.2 is the refractive index of Bi2212. Actually, for conventional Bi2212 mesas, the cavity quality factor *Q* ~ 10 is not as high as for quantum cascade lasers or solid state lasers, for example, so that *f* can be widely tunable^35^,^3^6,^43^.

Here, we demonstrate the performances of measurement systems that were not described previously^49^,^50^,^51^, detailing the characteristic features of the continuous monochromatic radiation from the IJJ-THz emitters. The first one is a compact and convenient THz imaging system cooled down to 40 K only by a Stirling cryocooler. The second one is a home-built Michelson type of interferometer constructed by modifying a reflection type of IJJ-THz emitter imaging system to develop a phase-sensitive imaging system. Finally, an IJJ-THz emitter device to measure the absorption coefficient of a liquid sample for temperatures between 0 °C and 70 °C is described in detail.

## Results and Discussion

The IJJ-THz emitting device is made from a piece of a single crystal of Bi2212 grown by a traveling-solvent floating-zone method. The crystal is annealed overnight at 550 °C in air and quenched to obtain a sharper superconducting transition behavior and the optimum doping level. The crystal is cut into a few mm in lateral dimensions, then is cleaved to a few *μ*m in thickness. The cleaved surface is immediately deposited with Ag and then Au layers 50–100 nm thick. A rectangular stand-alone mesa is made by Ar milling, mounted between two sapphire plates with deposited gold electrodes, and is placed in a Cu holder and sandwiched mechanically. More details of the stand-alone mesa sandwich fabrication are given in ref. 52.

### Compact and simple imaging system using a Stirling cryocooler down to 40 K

#### Measurement setup

**Figure 1:**
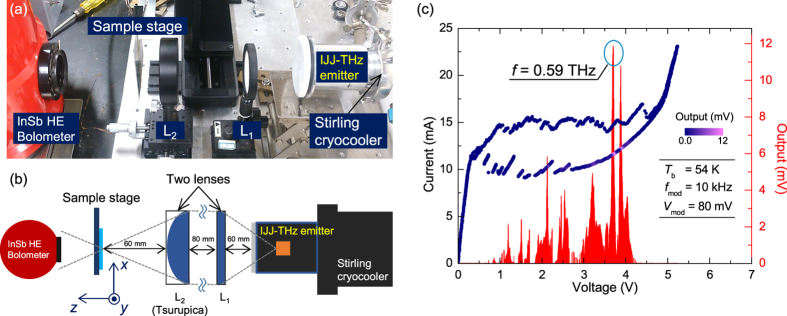
(**a**) A photograph and (**b**) a schematic diagram of the experimental setup. (**c**) The *I*-*V* characteristics of the IJJ-THz emitter used for this measurement. The data are plotted using the color code corresponding to the output voltage of the InSb HE bolometer, which is also plotted as a function of *V*.

Figure 1(a) shows a photograph of the experimental setup used for a compact and convenient imaging system cooled by a Stirling engine operated by a 45 V dc battery with a cooling power of better than 16 W at 77 K and an input power of 240 W, whose cold tip is cooled down to 35 K within a stability of 0.1 K. A corresponding schematic diagram of the experimental setup is shown in Fig. 1(b). The IJJ-THz emitter is fixed mechanically to the cold tip and can be cooled to 40 K within 30 minutes. The THz EMWs generated by the IJJ-emitter are collected and transformed into a parallel beam by the first lens, L_1_, then it is focused onto the sample by the second lens, L_2_, with a focal length of 60 mm. These two lenses have the same diameter of 52 mm. As seen in Fig. 1(a,b), the total length between the cryocooler and the sample is about 20 cm. Except for the detector, the total mass of this system is less than 4 kg.

The transmitted THz beam is detected by a high-speed InSb hot-electron (HE) bolometer, and the detection power is monitored by a lock-in amplifier with a time constant of 3 ms. In order to acquire two-dimensional (2D) images, the sample set at the L_2_ focal point was scanned in the *x* (horizontal) and *y* (vertical) directions at a variable speed below 130 mm/s with a 2D(*x*, *y*) scanning stage. The analog signal from the lock-in amplifier is converted to a digital signal with a resolution of 16 bits by an analog-to-digital (A/D) converter. The motion of the stage can be controlled by a homemade LabVIEW (National Instruments Co.) program. The actual signal processing of our systems is performed as follows: In order to generate the pulse-modulated (PM) THz wave, the IJJ-THz emitter was modulated with an ac square-wave voltage of amplitude ~80 mV and frequency 10 kHz onto the dc bias voltage with a function generator, where 10 kHz is the frequency of the optimal noise performance of InSb HE bolometer. We note that this electronic modulation detection method is superior to that of an optical chopper modulation, since the far-infrared background noise from the ambient spurious radiation can be completely rejected. The motivation for this detection method in a transmission-type imaging system was described previously^49^.

For this measurement, a stand-alone mesa sandwich IJJ-THz emitter of dimensions 58 × 350 × 4.7 *μ*m^3^ was used. Although this device is comparatively thick, as seen in Fig. 1(c), a large bias *V* can be applied to it without *I*-*V* characteristic back-bending, which led to negative dynamical resistance in earlier conventional mesas. The present stand-alone mesa sandwich structures have high heat-exhaust efficiency due to the surrounding sapphire and copper plates with high thermal conductivity. As a result, this stand-alone type of IJJ-THz emitter can generate radiation from 0.3 THz to 2.4 THz by changing the bias point and the bath temperature^44^,^46^,^52^. We note that the thicker mesa has more layers for THz radiation, so that it is in general expected to have stronger THz radiation. However, the thicker mesa may have more heat generated and could suffer from increased self-heating. It is empirically known that the optimal mesa thickness is a few *μ*m. The reproducibility of the stand-alone mesa has recently improved greatly to 20–25%.

An example of the *I*-*V* characteristics of the emitter at the bath temperature 54 K is shown in Fig. 1(c), where the output power detected by the HE bolometer with a modulation amplitude of *V*_mod_ = 80 mV at the frequency of *f*_mod_ = 10 kHz is also plotted as a function of *V*. This emitter generates 0.59 THz wave radiation at the bias point encircled by the solid curve, which was measured by using the interference pattern technique reported previously^49^. The detected power is estimated to be about 5.1 nW from the responsivity of the InSb HE bolometer of 3.3 mV/nW during the measurements.

#### Basic properties and demonstration

In an imaging system with a raster scan method, the beam waist (the minimum beam diameter at the focal point) and the depth of focus are important parameters for the optical system performance. The beam waist can greatly affect the spatial resolution of the images and the depth of focus indicates the tolerance of the spatial resolution to the optical axis direction changes.

In order to estimate these two parameters in our THz imaging system, we scanned an edge of a 1 mm thick metallic plate by changing the position of the optical *z* axis direction. The measured data were analyzed based on the knife-edge method. Assuming a Gaussian distribution of the beam’s intensity, the transmitted intensities *I*(*x*) obtained around the horizontal *x*-position can be fitted by the following equation:


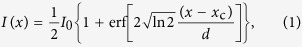


where *I*_0_ is the maximum intensity of the beam, *x* is the position of the knife edge, *x*_c_ is the distribution center, *d* is the full width at half maximum (FWHM) of the Gaussian distribution, and erf(*x*) is the error function.

**Figure 2:**
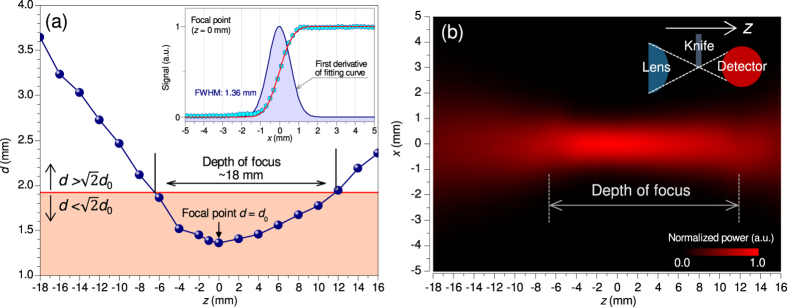
(**a**) Beam width *d* versus optical axis direction *z*. The inset show the measurement result at the focal point. The data fitted by the error function, Eq. (1) and its first derivative are also plotted. (**b**) Contour plot of the first derivative of the fitting curves.

Here, the beam diameter *d* is a parameter in Eq. (1). Figure 2(a) shows measured values of *d* at many positions in the *z* direction. The inset shows an example of the measured result at the focal point (*z* = 0). The fitting curve and its first derivative are shown in the inset in Fig. 2(a). The *I*(*x*) observed is fitted very well by Eq. (1). The beam waist *d*_0_ of our system is estimated to be 1.36 mm. The depth of focus Δ*z*, defined as the range where the beam diameter is smaller than 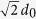, can therefore be estimated to be about 18 mm.

Δ*z* can also be calculated by the following relation as twice the Rayleigh range:


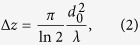


where *d*_0_ is the beam waist and *λ* is the wavelength of the EMWs. In our case, the value of the fitting *d*_0_ is 16.5 mm, which is consistent with its measured value of ~18 mm.

First derivatives of the fitting curve at each position are shown in Fig. 2(b) by the contour color plot. This graph displays the spatial distribution of the beam intensity, which drastically broadens outside of the depth of focus. This means that if the sample’s thickness or the optical axial position is larger than this range, the spatial resolution is quickly lost.

**Figure 3:**
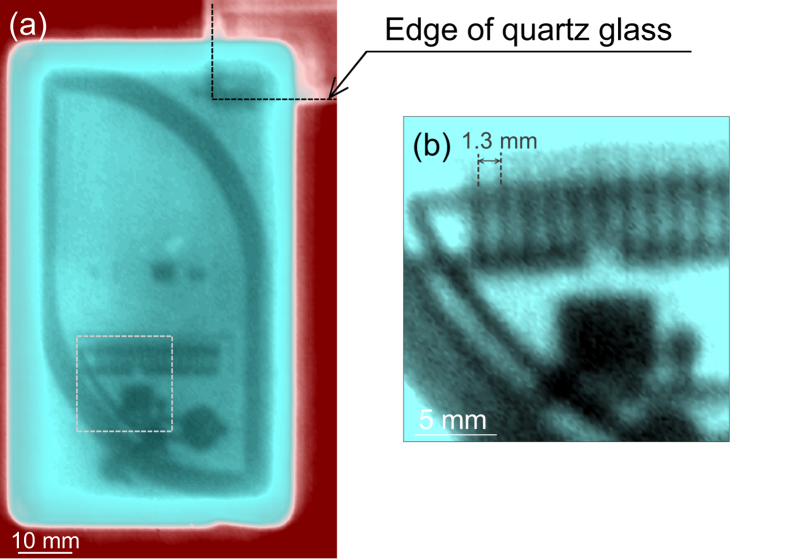
(**a**) Transmission-THz image of an IC card with scanning steps of 0.5 mm. (**b**) THz image scanned over the region enclosed by the dotted rectangle in (**a**) with scanning steps of 0.2 mm.

Next, in order to demonstrate the performance of the imaging system, we measured an IC card and the result is shown in Fig. 3, where the increment of the translation of the sample is 0.5 mm in Fig. 3(a). In Fig. 3(b), the region encircled by a doted box in Fig. 3(a) is displayed by remeasuring with a finner step of the increment of 0.2 mm.

As seen in both images, the circuit structures embedded inside the integrated-circuit (IC) card can be clearly resolved. Especially, the comb-shaped electric circuit structure with an interval of about 1.3 mm inside the plastic body can be clearly seen (see Fig. 3(b)). As a consequence, this THz imaging system can at least reach the spatial resolution of 1.3 mm. Here, we compare the image shown in Fig. 3 with the one given in Fig. 10 of ref. 4, which reported the performance of an imaging system using a BWO. The image resolution of the BWO system is better than that of our system because the beam waist of the BWO system is half that of our system, and the output power of the BWO (~15 mW at this frequency range) is much stronger than that of the IJJ-THz emitter. On the other hand the depth of focus of our system is longer than that of the BWO system. As an additional advantage of our system, THz imaging over a wide frequency range will be available in the near future, according to recent reports^44^,^4^6.

### Construction of a high-resolution spectrometer based on a Michelson type of interferometer

**Figure 4:**
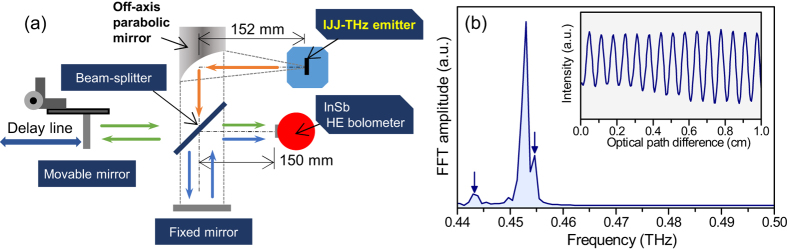
(**a**) Optical system of a FT-IR spectrometer based on a Michelson type of interferometer. (**b**) Spectrum obtained from FFT of the interferogram (inset) measured by the interferometer.

A Michelson type of interferometer, which allows one to measure the THz frequency spectrum of the radiation with sufficiently high resolution, can easily be constructed by modifying a reflection type of imaging system. Figure 4(a) shows the diagram of the optical system. A Si-wafer is used as a beam splitter as was done previously^50^. For the present measurements, the mirror was scanned over 18 cm (an optical path difference *L* = 36 cm) in 20 *μ*m steps and a scanning speed of 0.2 mm/s (15 min/scan). As a test operation, the THz wave emitted from the 80 × 400 × 2.4 *μ*m^3^ IJJ-THz emitter was used at the bath temperature of 45 K.

According to the principle of operation of this type of interferometer, the spectral resolution Δ*f* determined from Δ*f* = *c*/*L* is estimated to be 0.83 GHz. The spectrum obtained from the IJJ-THz emitter is displayed in Fig. 4(b) after performing a fast Fourier transform (FFT) of the interferogram, part of which is shown in the inset of Fig. 4(b).

In Fig. 4(b), it is noted that in addition to the main peak in the spectrum, two other small peaks can be seen near to 0.443 THz and 0.455 THz, as indicated by the vertical arrows. These peaks could not be resolved in previous measurements performed with a commercial spectrometer with a maximum resolution of 7.5 GHz, and suggest that the emission spectrum from the IJJ-THz emitter may not be monochromatic in this particular retrapping bias point region on the irreversible *I*-*V* curve, because the emission from this region is well known to be unstable due to the nonlinear ac-Josephson effect, resulting in the small splitting of the emission line as seen in higher resolution measurements. This is evidenced by wider spectral line widths previously observed for THz EMWs emissions in the retrapping region of the *I*-*V* characteristics^35^. Further spectroscopic studies with higher resolution are needed to better understand the IJJ-radiation.

By fitting the main peak with a Lorentzian function, the central frequency and the full-width at half-maximum are estimated to be 0.453 THz and 1.07 GHz, respectively. This spectral line width is close to the resolution limit of this system and similar to that of the Martin-Puplett type of interferometer used in ref. 25. Note that this value of the line width is much shorter and certainly is beyond the resolution limit of the commercial FT-IR spectrometer (FARIS-1, JASCO).

From the above, we conclude that a high-resolution FT-IR spectrometer based upon a Michelson type of interferometer was constructed successfully. Combining this interferometer with our imaging systems, a continuous-wave THz phase imaging system^53^ is now under construction.

### Measurement of the absorption coefficient of a liquid

THz EMWs are known to be strongly absorbed by liquid water in the wide frequency range from the microwave to the far infrared regions^54^. In aqueous solution, it is known that solute molecules and hydrated water show an interesting interplay of relaxational responses in the MHz to GHz spectral region^55^. However, because of the complicated behavior consisting of three relaxational responses of solute molecules, hydrated water, and bulk water, it is difficult to extract information on hydrated water from the low-frequency dielectric responses. On the other hand, the dielectric loss is simply reduced according to the amount of hydrated water in the THz region. Therefore, the hydrated state of a liquid sample can be evaluated by measuring the absorption coefficient of the sample.

A technique for measuring an absorption coefficient of a liquid sample was developed previously by using a wedge-shaped quartz cell (WSQ-cell)^4^,^4^9,^56^,^57^. In this method, the WSQ-cell is filled by the liquid and scanned linearly from the thinner end to the thicker end, and the transmitted THz intensity is measured as a function of the scanned length. The measured data is fitted by the equation *I* = *I*_0_ exp(−*αt*), where *I* is the intensity of the transmitted THz EMWs, *I*_0_ is the power of the THz EMWs, *α* is the absorption coefficient and *t* is the thickness of the sample. This WSQ-cell enables one to obtain the absorption coefficient very easily and precisely, even for a sample with a high absorptivity, because only a line scan is needed for the measurement, and parameters other than *α*, such as the absorption of the quartz plates, are usually negligible.

**Figure 5:**
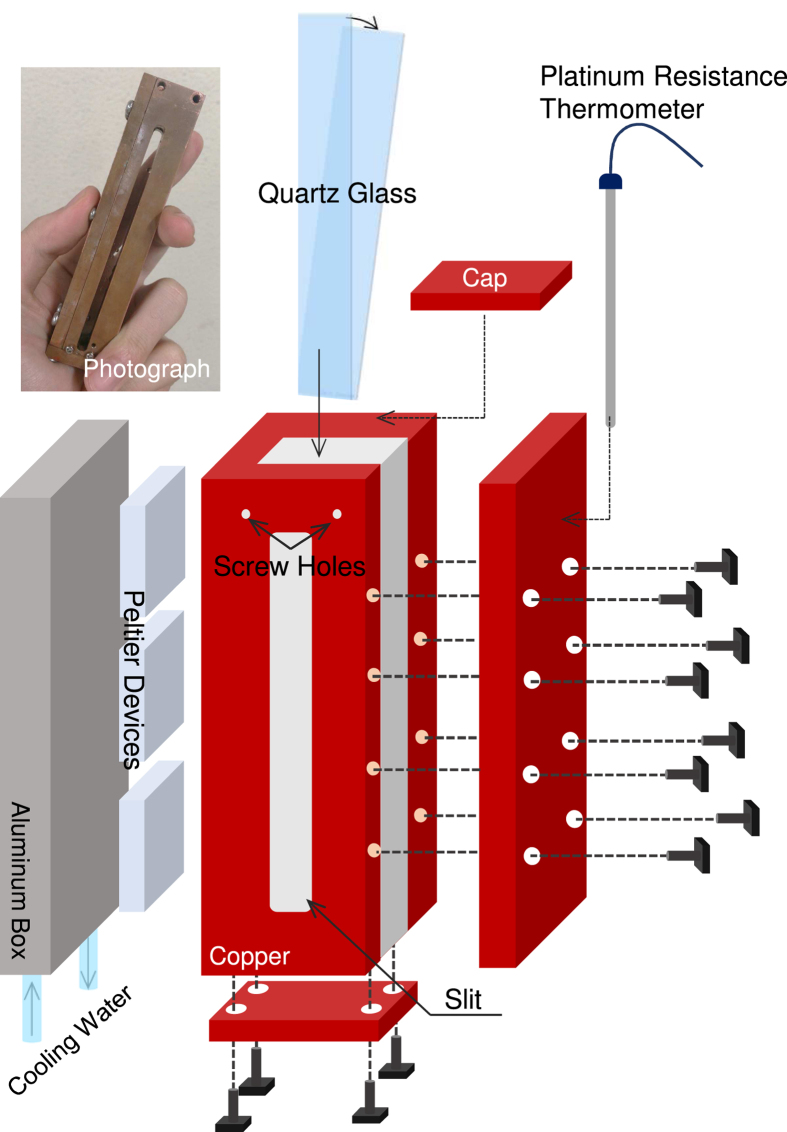
Schematic views of the variable-temperature WSQ-cell. An optical photograph is shown in the upper left.

Figure 5 is a schematic diagram of the variable-temperature WSQ-cell. Two quartz plates are fixed by screws into a wedge shape inside a copper box. The copper box has a slit through which the THz EMWs can pass. In order to control the sample temperature, four Peltier devices are set between a water-cooled aluminum box and a side of the copper box, as shown in Fig. 5.

A temperature controller is used to control the Peltier devices. In order to monitor the sample temperature, a platinum resistance thermometer was attached to a side wall of the copper box and connected to the temperature controller. The absorption coefficient of the measured liquid is calculated and plotted as a function of the sample temperature by a homemade LabVIEW program. This system can control the temperature *T* between 0 and 70 °C with an accuracy of ±0.5 °C.

**Figure 6:**
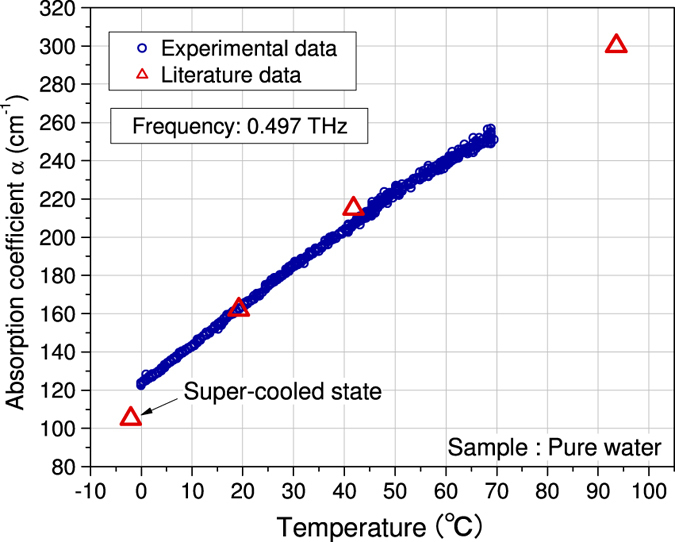
Temperature dependence of the absorption coefficient of pure water. Data from ref. 58 are also plotted for comparison.

To demonstrate the utility of this system, the temperature dependence of the absorption coefficient of pure water with a resistivity of more than 1 MΩ · cm at 25 °C (Kyoei Pharmaceutical Co., Ltd.) was measured. Figure 6 displays our results and compares them with other data obtained previously by a THz-TDS system (see ref. 58). A 60 × 400 × 1.6 *μ*m^3^ IJJ-THz emitter that generates 0.497 THz radiation is used for the present measurements. The measurements can be made within about 16 s per scan at a fixed *T* to obtain *α*(*T*). The measured *α*(*T*) agrees very well with other published data, as shown in Fig. 6.

The error bars of the measured *α*-values are ±0.6 cm^−1^ at 0 °C and ±3.9 cm^−1^ at 70 °C. These values provide the most accurate measurements of the absorption coefficient of water in this frequency region because the signal-to-noise ratio is much higher than for other techniques.

## Summary

We presented here three THz measurement systems for applications using a stand-alone mesa sandwich IJJ-THz emitter. First, a compact THz imaging system using a Stirling cryocooler with a measured beam waist and depth of focus was made, and to demonstrate its utility, it was used to obtain the transmission THz image of an IC card.

Second, a Michelson type of interferometer based on a reflection type of THz imaging system was shown. The interferogram was clearly observed, and its Fourier transform was made to obtain it frequency spectrum showed that device has a spectral resolution of 0.83 GHz which is much better than the resolution of the commercial FT-IR spectrometer (FARIS-1) used for previous works (~7.5 GHz).

Finally, we described a system to measure the absorption coefficient of a liquid sample at a variable temperature *T* between 0 and 70 °C within an accuracy of ±0.5 °C. The absorption coefficient *α*(*T*) of pure water was obtained with high accuracy, and is in good agreement with previous measurements.

## Methods

In the systems presented here, we used the following components: a Stirling cooler engine (CryoTel GT, Sunpower Inc.), the lens L_2_ shown in Fig. 1 (Terahertz-Super Lens “Tsurupica”, PAX Co.), a InSb HE bolometer(QFI/2BI, QMC Instruments Ltd.), a lock-in amplifier (Model 7265, EG & G instruments Ltd.), a 2D scanning stage (SGAMH26-200, Sigma Koki Co.), and A/D converter (PXI-5922, National Instruments), a function generator (33220A, Agilent), and a temperature controller (OCE-TCR24600WL, Ohm Electric Inc.) was used to control the Peltier devices.

## Additional Information

**How to cite this article**: Nakade, K. *et al.* Applications using high-*T*_c_ superconducting terahertz emitters. *Sci. Rep.* **6**, 23178; doi: 10.1038/srep23178 (2016).
